# Subjective learning gain from a simulation-based health management course: a mixed methods study

**DOI:** 10.3389/fpubh.2024.1400135

**Published:** 2024-10-01

**Authors:** Ren-Ping Gu, Fang Zhao, Jie Bai, Shan-Shan Sun, Ai-Yong Zhu

**Affiliations:** School of Nursing and Health Management, Shanghai University of Medicine and Health Sciences, Shanghai, China

**Keywords:** simulation-based education, healthcare, learning gain, health management, medical education

## Abstract

**Objectives:**

Simulations are increasingly being offered as part of the educational experience of healthcare students. We used a Health Management Scenario Simulation system to create a course. This study aimed to evaluate learning gains before and after the course.

**Methods:**

Based on the learning strategies of framing, simulation, and debriefing, the Health Management Scenario Simulation course lasted 4 weeks and was conducted online. Learning gain was assessed using a comparative self-assessment questionnaire administered electronically at the beginning and end of the course. We organized focus group interviews and collected quantitative data after students completed the simulations and the questionnaire. These data were subjected to descriptive statistical analysis and thematic grouping using frequency counting.

**Results:**

There were 195 health management students enrolled in the course. In total, 265 anonymously completed questionnaires were received, 141 (72.31%) on the pre-simulation and 124 (63.59%) on the post-simulation. All questionnaire item gain values were positive, except the item “I can identify common health risk factors,” which showed no change. The skills domain showed the highest learning gain, ranging from 16 to 22%. Six students participated in the focus-group study. The main themes that emerged from students’ reflections were learner-centeredness, competencies, and career development.

**Conclusion:**

Students acquired health management skills through the simulation, which contributed to the development of basic attitudes and skills in their professional careers. Students’ comments highlighted the value of practicing health management skills in a simulated environment.

## Introduction

Non-communicable diseases (NCDs) have become the leading cause of death and disease burden (1). For example, the total number of adults with diabetes in mainland China is estimated to be 129.8 million, accounting for 24% of adults with diabetes worldwide in 2017 (2), and this is projected to increase rapidly to 202.84 million by 2050 (3). Owing to the increased burden created by NCDs, the Chinese State Council issued guidelines in 2015 for establishing a tiered medical service system. Under this hierarchical healthcare delivery system, primary healthcare providers are the first point of contact for patients and are responsible for providing health promotion and prevention, as well as health management and maintenance of chronic diseases (1). The health management provided by primary healthcare providers includes comprehensive monitoring, analysis, evaluation, planned prevention, and various risk factor interventions for individuals or groups (4). Therefore, these are the core skills health management undergraduates need to master. However, during undergraduate teaching, these skills are scattered across different courses (5). In addition, the long-term nature of NCD health management restricts these undergraduates’ opportunities to acquire the skills required (6).

As a way to improve the opportunities for skill acquisition, simulations are used to improve the healthcare educational experience. Simulation-based learning can be classified into role-playing, physically based, and computer-based simulation (7). Computer-based simulations have been shown to support the rapid acquisition of clinical skills, clinical decision-making, clinical reasoning, and non-technical skills (e.g., interpersonal communication, conflict management, and decision-making under pressure) (8–11). Such simulations can be conducted at any time and repeated as required; therefore, students can refine their skills and address errors made in each iteration (11, 12).

Computer-based simulation tools have been introduced in health management education in recent years. In an advanced health appraisal course, the integration of virtual clinical simulations has been proven to be a feasible, cost-effective strategy for providing an additional means for student skill acquisition and evaluation (13). In one study, a virtual gaming simulation was implemented in a health assessment class. The results indicated that virtual gaming simulations could provide experiential learning opportunities that promote engagement and allow learners to acquire and apply new knowledge while practicing skills in a safe and realistic environment (14). Wright et al. (15) applied virtual simulation to nursing care education of patients with diabetes. The findings indicated that it was a positive experiential learning endeavor and helped students understand adult health concepts. With the development of digital technology, the integration of computer-based simulation instruction in health management education has become an inevitable trend. However, it remains necessary to integrate all health management courses in simulation-based education.

Based on the advantages of computer-based simulations, this study used a Health Management Scenario Simulation (HMSS) system. Acting as community health managers, students conduct situational health management for individual residents based on visual data and textual background information. They explore health-risk issues for individuals and community populations, provide health guidance, and develop personalized health intervention plans. They also arrange reasonable follow-ups for health monitoring, adjust intervention plans according to changes in indicators, and collaborate with multiple institutions to jointly promote the health level of the simulated case subjects. After selecting a specific chronic disease, the system randomly selects a case from a patient database. Starting from the first contact with the patient, the system sets up questions. Once the student solves this problem, the next step in the health management service process presents itself. The student comprehensively analyzes and evaluates the key patient information based on previous information until all health service process modules are completed. However, the benefits of introducing simulation training using HMSS, have not yet been investigated. The aim of this study was to evaluate the learning gains before and after the course. Specifically, we aimed to understand how the use of Health Management Scenario Simulation could support the development of health management knowledge, skills, and attitudes, through comparative self-assessment.

## Methods

### Setting

The four-week HMSS course was conducted online. The learning strategy followed the recommended three-phase approach of framing, simulation, and debriefing (16). In Week 1, students were given general instructions about the training simulation in the form of pre-recorded lectures, and an online live class was held to help them practice the simulation platform. Over the next 2 weeks, students engaged in simulation activities in which a group of three students worked together to perform the entire health management process. During this period, students had to complete six cases. Each case consisted of seven modules: health information collection, assessment, goal setting, intervention, follow-up, evaluation, and emergency response. Students were required to select the most relevant items for the case in each learning module. During the simulation, students did not receive any support from trainers. After the simulation, the HMSS generated a raw score based on the correctly chosen relevant items. Following the simulation in Week 4, students were debriefed and engaged in critical reflection with their peers and teachers.

### Participants

Third-year undergraduate Bachelor of Health Management students from 65 universities in the Chinese Health Management Professional Alliance participated in this study. Before the study, the participants had completed all of their health management curriculum and possessed basic knowledge about health management. Because the participants engaged in small-group learning, a minimum of three students was required from each university. A total of 195 third-year health management students were included in the study.

### Study design

We conducted a mixed methods study to assess the learning gains of students participating in an HMSS course. This study was approved by the Institutional Ethics Review Board of Shanghai University of Medicine & Health Sciences (2024-zxkt-01-310225198301136663). Participation in the study was voluntary and written informed consent was obtained from every study participant. Subsequently, students received a message invitation to complete an anonymous pre-questionnaire via sojump.^1^

The four-week HMSS course was provided to all participants. The course started in December 2024. In Week 1, a framing was held to familiarize students with the cases before the simulation began. During the next 2 weeks, the students were required to complete six case scenarios. In Week 4, the students were divided into 10 groups, each with an assigned teacher who organized debriefing sessions with the students.

A post-questionnaire was administered electronically at the end of the course. Immediately after the students had completed the questionnaire, interested students were invited to participate in a face-to-face focus group.

### Quantitative

#### Instruments

Learning gain was assessed using comparative self-assessment (CSA) (17), which integrates specific learning objectives into a program evaluation tool. Raupach et al. (17, 18) developed CSA by comparing initial and final self-assessments and adjusting the difference for initial performance, and found that it was highly correlated with actual learning outcomes measured in objective exams. CSA is a valid tool for assessing the actual increase in competence or knowledge as it accounts for the initial level of participants’ competence or knowledge. This makes it superior to plain mean differences and effect sizes in detecting high-quality teaching as well as curricular shortcomings. In addition, CSA adequately reflects objectively measured performance levels and is responsive to changes in the curriculum (17). This tool yields learning outcome data ranging from −100 to +100%. In this study, CSA comprised nine items (see Table 1) and addressed all domains of medical education, that is knowledge, skills, and attitudes (KSAs) (17, 19), which serve as fundamental metrics for evaluating competencies, performance, and outcomes (20, 21). These items were adopted from previous studies and were discussed among an expert group selected based on their expertise. Each item was scored on a six-point Likert scale ranging from 1 (strongly agree) to 6 (strongly disagree). The item-specific CSA gain (%) was calculated using the following formula (17, 22):


$$CSA\;\mathrm{gain}\;\left(\%\right)=\frac{\mu_{pre}-\mu_{\mathrm{post}}}{\mu_{pre}-1}\times 100$$

**Table 1:** Subjective learning gain reported by the undergraduates.

Items	$\mu_{pre}$ (SD)	$\mu_{\mathrm{post}}$ (SD)	CSA gain (%)
Knowledge			
1. I can identify common health risk factors.	2.16 (0.83)	2.16 (0.83)	0.00
2. I feel confident in assessing health status.	2.30 (0.89)	2.19 (0.78)	9.24
3. I am familiar with the basic theories, models, and methods of health intervention.	2.48 (1.05)	2.27 (0.79)	16.54
Skills			
4. I feel confident in collecting health information.	2.43 (1.00)	2.23 (0.85)	16.26
5. I feel confident in applying health risk assessment tools.	2.52 (0.98)	2.24 (0.82)	22.58
6. I feel confident in developing reasonable health intervention plans.	2.42 (0.93)	2.19 (0.77)	19.33
Attitudes			
7. I am aware of the professional nature of the Health Service and Management major.	2.32 (0.87)	2.14 (0.74)	15.79
8. I feel confident in becoming a future professional.	2.16 (0.88)	2.15 (0.77)	0.87
9. I am familiar with the core content of health services and management.	2.46 (0.95)	2.22 (0.81)	19.67

CSA, comparative self-assessment.

where $\mu_{pre}$ is the group mean initial self-assessment and $\mu_{\mathrm{post}}$ is the group mean self-assessment after the course. Learning gain was assessed using an anonymous online CSA questionnaire. The sample size was calculated with the assumptions of increasing learning gain regarding the HMSS course, as per the CSA questionnaire by 20% with a power of 80%, and a two-sided test at a 0.05 significance level. We will recruit a sample of 101 participants.

#### Data analysis

Statistical analyses were performed using IBM SPSS Statistics for Windows, version 21.0. Demographic characteristics and CSA questionnaires were analyzed using descriptive statistics.

### Qualitative

#### Data collection

The focus group (FG) used a pretested semi-structured interview guide (Appendix 1). After informed consent was obtained, an interview method with open-ended questions focusing on the study objectives was conducted. Open-ended questions allow space for longer narratives and information from respondents. According to the respondents’ responses, the interview process aims to achieve the main objectives of the research. The FG interview lasted between 30 and 40 min and had six participants. Students were told that the interview would be recorded and transcribed but that no individual contributor would be identified.

#### Data analysis

The information obtained from the FG interview was transcribed and analyzed using qualitative text analysis in a five-step process by two investigators (FZ and JB) (23). The first step was to prepare the data and obtain an overview by listening to and reading the interviews intensively. Second, we identified the main categories corresponding to the questions asked during the interviews. Third, we coded the data according to the main categories. Next, the text passages of the main categories were compiled, subcategories were formed inductively from the material, and text passages were assigned to the subcategories. Finally, the category-based analyses and results were presented. All the interviews were transcribed verbatim. The first step was independently conducted by FZ and JB. They conducted step 2 in collaboration. In step 3, FZ and JB separately coded the data according to the main categories of two selected interviews. They discussed the discrepancies in order to achieve an accurate assignment. FZ coded the remaining transcribed interviews, which were then discussed with JB. In the next step, subcategories were formed by FZ and discussed with JB. Finally, FZ conducted step 5. We used MAXQDA software to organize and structure the data.

## Results

### Quantitative

There were 195 health management students enrolled in the HMSS course. In total, 265 (women = 186, 70.19%; men = 79, 29.81%) anonymously completed CSA were received. Of these, 141 (72.31%) responses were received on the pre-simulation CSA and 124 (63.59%) on the post-simulation. No records were excluded because of missing data. The study participants averaged 20.27 years old (SD = 1.15).

All CSA gain values were positive, except the item, “I can identify common health risk factors,” which showed no change, but the actual degree of gain varied greatly from item to item (Table 1). The skills domain showed the highest learning gain, ranging from 16 to 22%. The HMSS implements the entire process of health management, so it helps to nurture necessary health management skills. The statement “I feel confident about becoming a future professional” yielded only a negligible gain. This may be because they had not yet had contact with real patients and were thus unable to compare the simulation with actual situations and make relevant judgments. Overall, the highest learning gains were reported for Items 5, 6, and 9.

### Qualitative

Six students (three women, three men) aged between 21 and 22 participated in the focus-group study which was conducted face to face. Three main themes emerged from the FG interview analysis: learner-centeredness, competencies, and career development (see Table 2).

**Table 2:** Themes and sub-themes and the frequency with which participants expressed them.

Themes	Sub-themes	Frequency
Learner-centeredness		
	Actively exploring	6
	Self-directed learning	4
	Interest in learning	3
Competencies		
	Knowledge application	9
	Team collaboration and communication	8
	Dealing with unexpected events	5
	Skills training	5
	Decision-making	3
Career development		
	Preparing for career	5
	Professional Identity	3

#### Learner-centeredness

Students described the HMSS course as an independent, self-directed environment and a way to learn more actively without feeling the psychological pressure of managing patients’ expectations. This is shown as expressed by our participants:

S5: “*After several simulations, we have a better understanding of the case. We can determine the disease characteristics by reading the community profile. The community profile during the simulation showed that the prevalence of obesity, diabetes, and hypertension in the community was higher than the average level in China, so we can understand the main health issues affecting the community population.”*

Students emphasized how the simulation addressed learning gaps that were not met using traditional classroom methods (such as lectures or textbook reading) and that it can serve as a supplement to traditional teaching methods and activities. This is shown as expressed by our participants:

S2: “*Our autonomy and exploratory ability are gradually increasing. Compared to the dull and tedious knowledge in the traditional classroom, this course is more innovative, which can attract students’ interest in the classroom and increase their autonomy in learning.”*

#### Competencies

All participants mentioned the competencies they had acquired, both technical and non-technical. Technical competencies include knowledge acquisition and skills training. This is shown as expressed by our participants:

S1: “*I have learned how to apply theoretical knowledge of health management in real-life scenarios and improved my skills regarding crisis management and team collaboration.”*

Non-technical competencies that can be addressed by the course include teamwork, communication, and decision-making. This is shown as expressed by our participants:

S2: “*Conflicts of opinions among group members were resolved through mutual communication and agreement.”*

#### Career development

The course applies simulation learning to improve practice, contributing to the development of basic attitudes and skills for professional careers. Simulation integrates health information collection, health-risk assessment, health intervention, and follow-up services into a health management virtual simulation experiment of chronic diseases so that students have an intuitive and systematic understanding of the health management service process, which is particularly useful at the start of their professional life. This is shown as expressed by our participants:

S3: “*The course greatly enhances my practical operating and emergency response abilities, which is extremely important for my future career. Through such practical experience, I can better prepare for the challenges that may arise in a real work environment. This not only enhances my professional skills, but also deepens my understanding of the health management industry, thereby enabling me to more effectively apply the knowledge and skills I have acquired in my future work.”*

The students gave a positive evaluation of their profession and assessed the importance of their professional roles in their self-identity. This is shown as expressed by our participants:

S2: “*This makes me better understand and identify with the health management professional, as well as work content*.”

## Discussion

We analyzed the subjective learning gain both descriptively and qualitatively, demonstrating positive outcomes from the majority of the participants. In the qualitative analysis, the skills domain showed the highest learning gain. Quantitative results were also obtained, emphasizing the positive effects of improving health management skills. Similar studies have also found that simulation-based education can help medical and nursing students, as well as healthcare professionals, develop core skills required for clinical practice (8, 24, 25). Most health management education programs require students to complete internships in a healthcare setting to gain practical experience and integrate their knowledge and health management skills (5). During this transition, owing to the contradiction between the long-term nature of chronic disease health management and the short-term nature of internship placements, students often lack confidence, which affects their ability to work in complex healthcare fields (6). Simulation-based education is an effective and efficient way to acquire the necessary skills. It places health management students in the position of health managers, thereby nurturing the necessary competencies and skills for assessment, intervention skills, follow-up management, and decision-making (26, 27).

Our study showed that the HMSS course has a limited impact on knowledge acquisition, as students reported few learning gains in their health assessment knowledge. Similarly, in a quasi-experimental study at Ohio University, there was no significant difference in the course grades between the intervention and control groups following the addition of virtual clinical simulations into an Advanced Health Appraisal course (13). This suggests that the HMSS’s function needs to be further expanded in terms of knowledge. The second-generation system will add a knowledge dictionary that allows access to core knowledge related to health management.

Regarding career development, students reported that the HMSS course contributes to the development of basic attitudes and skills for professional careers. For medical students, the appraisal of skills against specialty is a crucial factor among multiple factors that stimulate the choice of a particular specialty (28). In a study in Australia on factors that affect doctors’ choices of a generalist or specialist medical career, receiving feedback and endorsement of focused skills was related to choosing to become a specialist. This occurred at a stage in which they were impressionable and open to new experiences (29). The HMSS course provides health management students with the opportunity to evaluate and develop their professional skills. Specifically, the course integrates all health management courses and implements the entire process of health management. This contributes to the development of a more robust health management workforce.

We also showed that our simulation training may have benefited from the small group environment because when asked about the difficulties encountered during training, three students mentioned conflicts between team members that they encountered at the beginning of the team’s training. Later, during this process, they gradually strengthened their communication skills and resolved conflicts. This is beneficial because studies have shown that small-group training facilitates active learning, enhances deep learning, and develops communication skills and conflict management (30, 31).

In this study, students indicated that the HMSS provided an independent, self-directed learning environment that can serve as a supplement to traditional teaching methods and activities. For medical students, instructional strategies should encourage them to engage in self-directed learning (32), where they exert control over their own learning and direct strategies to manage their learning tasks. Self-learning methodologies in simulated environments encourage students to be the protagonists in their learning processes (33, 34). Based on the simulated operational steps, students can effectively exert their autonomous perception abilities and reinforce their knowledge and competencies (35). In future practice, health management educators could incorporate the HMSS into their teaching curriculum.

Students also reported a better understanding of the health management process after several simulations. Constructivist concepts highlight the importance of repeated interactions between learners and the object of learning, and the importance of others in the process of knowledge construction (36). Simulation-based education is not limited by time or space. Students can implement health management in controlled settings, where it is possible to make mistakes and learn from errors with no consequences for the integrity of real patients (37). In addition, the possibility of timely feedback and repetition allows them to reassess information and adjust their intervention plans. This should promote the development of health management skills before they begin directly serving patients (11). Thus, simulation can be an effective learning strategy for skills training.

### Limitations

One limitation of this study was that those interested in simulation education were more likely to participate, which may have resulted in a selection bias. Another issue is that data were collected using a self-report survey, which is not always an accurate indicator of actual competence. The response rates for the pre- and post-simulation quantitative surveys were 72.31 and 63.59% respectively, raising the possibility that some of those failing to reply after the simulation were students who were less successful and consequently less willing to respond. While the comparative aspect of this study is somewhat limited by the fact that the data gathered before and after the simulations were not from identical groups, the data are reliable because the CSA’s ability to assess learning gain compares favorably with more traditional measures of pre- and post-differences (17). Finally, although behavior patterns in simulation are similar to those in real-life situations, the transfer of knowledge from the simulator to the clinical setting with actual patients is always a challenge (38).

## Conclusion

This study evaluates learning gains using Health Management Scenario Simulation system to teach students in China. Students acquired health management skills through the small-group simulation, which contributed to the development of basic attitudes and skills in their professional careers. Students’ comments highlighted the value of practicing health management skills in a simulated environment. The HMSS, integrating with self-directed learning environment, can serve as a supplement to traditional teaching methods and activities. Future research should address problems associated with low response rates. In addition, studies should include a control group for comparison and examine the influence of simulation training on developing long-term competencies. Furthermore, future research should measure actual outcomes to examine the relationship between computer-based simulations and students skill acquisition.

## Data Availability

The original contributions presented in the study are included in the article/Supplementary material, further inquiries can be directed to the corresponding author.

## Footnotes

^1^http://www.sojump.com
