# Supplementary material for: Subjective learning gain from a simulation-based health management course: a mixed methods study
Source: Front Public Health. 2024 Oct 1;12:1400135. doi: 10.3389/fpubh.2024.1400135 (PMC11473370; doi:10.3389/fpubh.2024.1400135)
Supplement: Supplementary file 1 [file Presentation_1.pdf]

Focus group semi-structured questions.
(1) What did you feel about using the HMSS? Please specify?
(2) What difficulties did you encounter in the simulation? How did you overcome the difficulties?
(3) What did you take from experience in the simulation to your professional world? Please specify?
(4) Compared to traditional learning in the classroom, how would you define the experience?
(5) What about the contribution of the simulation in preparing you for your future workplace and professional roles?
